# Why local guidelines?

**DOI:** 10.4103/0974-7796.78547

**Published:** 2011-03

**Authors:** Shouki Bazarbashi

**Affiliations:** Oncology Centre, King Faisal Specialist Hospital and Research Centre, Riyadh, Saudi Arabia E-mail: bazarbashi@gmail.com

In this issue of the journal we present the locally agreed management guidelines of the four most common urologic cancers: renal cell cancer, bladder cancer, prostate cancer and testicular cancer.

Medical practitioners in Saudi Arabia are either multicultural or trained in different parts of the world. This usually results in different treatment approaches and management plans for patients of similar problems. Under the direction of the Saudi Oncology Society, a committee of experts from the medical oncology, urology and radiation oncology was established.

The mission of the genitourinary guidelines committee was to oversee the development of guidelines for the common genitourinary cancers that will improve the practice of medicine for urologic cancers in Saudi Arabia and to establish minimum recommendations that can be used by health authorities in their decision making when coming across cancer management. This committee is one of the different committees that was established for the development of management guidelines in all cancer sites, of which the first to be published was the lung cancer guidelines.[1] Ongoing goals of the committee are to improve the dissemination of these guidelines, ensure annual updates and act as a nucleus for collaborative research.

Members of the genitourinary guidelines committee represented major institutions from all different parts of the kingdom and are listed in Table 1.

**Table 1:** Genitourinary guidelines members and their affiliations

Shouki Bazarbashi	Oncology Centre, King Faisal Specialist Hospital and Research Centre	Riyadh
Abdullah M. Al Ghamdi	Department of Urology, Riyadh Military Hospital	Riyadh
Ahmed Salah	Prince Faisal Bin Bandar Cancer Center	Buraidah
Ali Aljubran	Oncology Centre, King Faisal Specialist Hospital and Research Center	Riyadh
Amjad Rehman	Department of Medicine, Aseer Central Hospital	Abha
Asharaf Abu Samra	Department of Urology, King Khalid National Guard Hospital and Princess Norah Oncology Center, King Abdulaziz Medical City	Jeddah
Baher Kamal	Department of Urology, University of Dammam	Dammam
Esam Murshid	Department of Oncology, Riyadh Military Hospital	Riyadh
Eyad Al Saeed	Department of Medicine, King Khaled University Hospital	Riyadh
Hussein Al Kushi	Prince Nora Oncology Center, King Abdulaziz Medical City	Jeddah
Ibrahim A. Al Oraifi	Department of Urology, King Fahad Specialist Hospital	Dammam
Jalal Al Shareef	Department of Urology, Alhada Military Hospital	Altaef
Jamal-Eddin Zekri	Department of Oncology, King Faisal Specialist Hospital and Research Center	Jeddah
Khalid Al Ghamdi	Department of Surgery, Security Forces Hospital	Riyadh
Khalid Al Othman	Department of Urology, King Faisal Specialist Hospital and Research Center, Alfaisal University	Riyadh
Khalid Balaraj	Oncology Centre, King Faisal Specialist Hospital and Research Center	Riyadh
Mahmoud Zakaria Al Ali	Department of Surgery, North West Armed Forces Hos	Tabuk
Mohammed Al Otaibi	Department of Urology, King Faisal Specialist Hospital and Research Center	Riyadh
Mohamed El-Naghy	Department of Oncology, National Guard Hospital, King Abdulaziz Medical City	Riyadh
Yasir Bahadur	Department of Oncology, King Abdelaziz University Hospital	Jeddah
Danny Rabah	Department of Urology, King Saud University	Riyadh
Naser Albugamy	Department of Urology, National Guard Hospital, King Abdulaziz Medical City	Riyadh

The committee agreed to use the established bulleted format used by the lung cancer committee. The committee nominated different members to present a draft of the guidelines on each cancer site that takes into account available evidence for each item. The draft is finalized during one or two sessions. Each recommendation is either referenced and level of evidence is indicated. Final draft was circulated for approval by all members.

The committee also agreed to use the evidence level (EL) categories used by the lung cancer committee which are summarized as follow:

1. EL-1 (high level): well-conducted phase III randomized studies or meta-analysis
2. EL-2 (intermediate level): good phase II data or phase III trials with limitations
3. EL-3 (low level): observational/retrospective studies/expert opinion

Finally, committee members agreed that clinical research is integral part of patients care and is the only way to advance and improve patient care, increase cure rate and decrease the presence of adverse effects from medical and surgical therapies.
